# Non-targeted profiling of semi-polar metabolites in Arabidopsis root exudates uncovers a role for coumarin secretion and lignification during the local response to phosphate limitation

**DOI:** 10.1093/jxb/erv539

**Published:** 2015-12-17

**Authors:** Jörg Ziegler, Stephan Schmidt, Ranju Chutia, Jens Müller, Christoph Böttcher, Nadine Strehmel, Dierk Scheel, Steffen Abel

**Affiliations:** ^1^Department of Molecular Signal Processing, Leibniz Institute of Plant Biochemistry, D-06120 Halle (Saale), Germany; ^2^Department of Stress and Developmental Biology, Leibniz Institute of Plant Biochemistry, D-06120 Halle (Saale), Germany; ^3^Institute of Biochemistry and Biotechnology, Martin Luther University Halle-Wittenberg, D-06120 Halle (Saale), Germany; ^4^Department of Plant Sciences, University of California-Davis, Davis, CA 95616, USA

**Keywords:** *Arabidopsis thaliana*, coumarin, hydroponic system, lignification, metabolite profiling, oligolignol, phosphate starvation, root exudates.

## Abstract

Plants have evolved two major strategies to cope with phosphate (Pi) limitation. The systemic response, mainly comprising increased Pi uptake and metabolic adjustments for more efficient Pi use, and the local response, enabling plants to explore Pi-rich soil patches by reorganization of the root system architecture. Unlike previous reports, this study focused on root exudation controlled by the local response to Pi deficiency. To approach this, a hydroponic system separating the local and systemic responses was developed. *Arabidopsis thaliana* genotypes exhibiting distinct sensitivities to Pi deficiency could be clearly distinguished by their root exudate composition as determined by non-targeted reversed-phase ultraperformance liquid chromatography electrospray ionization quadrupole-time-of-flight mass spectrometry metabolite profiling. Compared with wild-type plants or insensitive *low phosphate root 1 and 2* (*lpr1 lpr2*) double mutant plants, the hypersensitive *phosphate deficiency response 2* (*pdr2*) mutant exhibited a reduced number of differential features in root exudates after Pi starvation, suggesting the involvement of *PDR2*-encoded P5-type ATPase in root exudation. Identification and analysis of coumarins revealed common and antagonistic regulatory pathways between Pi and Fe deficiency-induced coumarin secretion. The accumulation of oligolignols in root exudates after Pi deficiency was inversely correlated with Pi starvation-induced lignification at the root tips. The strongest oligolignol accumulation in root exudates was observed for the insensitive *lpr1 lpr2* double mutant, which was accompanied by the absence of Pi deficiency-induced lignin deposition, suggesting a role of LPR ferroxidases in lignin polymerization during Pi starvation.

## Highlight

Root exudate metabolite profiling suggests antagonistic roles for individual coumarins during phosphate and iron deficiency. Oligolignol accumulation and root lignification indicate impaired oligolignol polymerization in local phosphate deficiency response mutants.

## Introduction

Although soils may contain high amounts of the element phosphorus, the concentration of soluble, inorganic phosphate (Pi) is rather low in the rhizosphere. As plants can only use Pi as a nutrient, either in its HPO42– or HPO4– form, they are constantly challenged by suboptimal Pi availability. As such, Pi is one of the most limiting nutrients for plant growth, and plants typically suffer from Pi deficiency unless mitigated by P fertilization (Raghothama, 1999; Hinsinger, 2001; Vance *et al.*, 2003; Abel, 2011; Chiou and Lin, 2011). To cope with limited Pi supply, plants have evolved several strategies, which include metabolic and biochemical adjustments for optimized Pi utilization and recycling, as well as mechanisms for increased acquisition from Pi-poor soils.

One strategy, defined as the local response, is characterized by reorganization of the root system architecture (Thibaud *et al.*, 2010). Under Pi deficiency, plants such as *Arabidopsis thaliana* develop more lateral roots and root hairs at higher density. Furthermore, this reorganization is accompanied by the arrest of root growth at the primary root tip (López-Bucio *et al.*, 2002; Ward *et al.*, 2008; Abel, 2011; Péret *et al.*, 2011; Müller *et al.*, 2015). This developmental plasticity enables the plant to better explore Pi-enriched soil patches in otherwise Pi-deplete soils. This phenotypic response is modulated by Fe availability and localized Fe accumulation in root meristems (Svistoonoff *et al.*, 2007; Ward *et al.*, 2008; Müller *et al.*, 2015). Two major players in the local response are *PHOSPHATE DEFICIENCY RESPONSE 2* (*PDR2*) and *LOW PHOSPHATE ROOT 1* (*LPR1*), which genetically interact and play roles in the secretory pathway (Ticconi *et al.*, 2009). Root development of the *pdr2* mutant exhibits hypersensitivity to low Pi, showing accelerated primary root growth arrest upon Pi deficiency (Ticconi *et al.*, 2004). *PDR2* codes for the single P5-type ATPase, AtP5A, of unknown transport specificity, which is localized to the endoplasmic reticulum (Jakobsen *et al.*, 2005; Ticconi *et al.*, 2009). On the other hand, *lpr1 lpr2* double-mutant plants are insensitive to low Pi, which is manifested by similar primary root growth under +Pi and –Pi conditions (Svistoonoff *et al.*, 2007). LPR1 and its close paralog LPR2 belong to the large family of multicopper oxidase, possess ferroxidase activity, and reside in the apoplast (Müller *et al.*, 2015). The *pdr2* and *lpr1 lpr2* mutants are largely but oppositely affected in the local root growth response to Pi limitation.

The systemic response, which occurs throughout the plants, mainly entails metabolic adjustments for more efficient Pi use and Pi recycling under Pi-deficient conditions, as exemplified by increased synthesis of sulfolipids relative to phospholipids, or changes in the levels of amino and organic acids (Raghothama, 1999; Hernández *et al.*, 2007; Morcuende *et al.*, 2007; Péret *et al.*, 2011; Pant *et al.*, 2015). Increased organic acid exudation into the rhizosphere, which promotes Pi solubilization in soil, has been reported for several plant species (Hoffland *et al.*, 1989; Neumann and Römheld, 1999; Hinsinger, 2001; Meyer *et al.*, 2010). As the link between increased organic acid exudation and better Pi uptake has been well established, most studies on metabolite profiling have focused on polar metabolites (Dinkelaker *et al.*, 1989; Zhang *et al.*, 1997; Pearse *et al.*, 2007; Tawaraya *et al.*, 2014; Valentinuzzi *et al.*, 2015). However, semi- and apolar metabolites have rarely been analyzed. Also, the majority of polar metabolites considered in previous investigations are mainly subject to control by the systemic response regulator PHR1 (Bustos *et al.*, 2010; Pant *et al.*, 2015). In contrast, Pi deficiency-induced changes in metabolite levels under control of the local response have not yet been reported. This paucity is explained by the currently available hydroponic systems, which do not allow the separation of systemic and local Pi deficiency responses (Neumann and Römheld, 1999; Pearse *et al.*, 2007; Agrawal *et al.*, 2012; Chaparro *et al.*, 2013; Ding *et al.*, 2013; Tawaraya *et al.*, 2014). Therefore, a possible relationship between Pi deficiency-induced alteration of root exudation and modification of root system architecture cannot be revealed unless systemic and local responses are unraveled.

We conducted this study to investigate changes in root exudate metabolite profiles that are specific for the local Pi deficiency response. Toward this goal, we developed a novel hydroponic system, which allows the separation of local and systemic responses. In addition, our system is suitable for liquid chromatography mass spectrometry (LC-MS)-based non-targeted and targeted profiling of semi-polar metabolites of root exudates. To identify Pi-related and root phenotype-specific exudate metabolite profiles, we compared three genotypes with different sensitivities to Pi deficiency-induced primary root growth inhibition, the wild-type (Col-0) reference, the hypersensitive *pdr2* line, and the insensitive *lpr1 lpr2* double mutant (Müller *et al.*, 2015). The data revealed profound and genotype-specific changes in root exudate composition upon Pi deficiency. Based on the characterization of several metabolites, we detected a partial overlap between Pi and Fe deficiency-induced changes in root exudate composition. Furthermore, an inverse correlation between oligolignol content in root exudates and lignin deposition in roots implicates a role of lignification in root system architecture modification during the response to external Pi limitation.

## Materials and methods

### Reagents and standards

All buffer constituents and solvents were of reagent or HPLC grade and obtained from either Roth (Karlsruhe, Germany), Sigma-Aldrich (St Louis, MO, USA) or J. T. Baker (Deventer, The Netherlands). Coumarin standards were purchased from PhytoLab (Vestenbergsgreuth, Germany; scopolin, isofraxidin, fraxin), and Sigma-Aldrich (esculin, esculetin, fraxetin, 4-methyl-umbelliferon, scopoletin). [2,2,4,4-^2^H]citric acid and [2,3,3-^2^H]malic acid were from CDN isotopes (Point Claire, QC, Canada), [2,2,3,3-^2^H]succinic acid and methoxyamine HCl from Sigma-Aldrich, and Silyl-991 from Macherey-Nagel (Düren, Germany).

### Plant lines and growth media

*A. thaliana* accession Columbia (Col-0) and the mutant lines *pdr2* and *lpr1 lpr2* (Col-0 background) have been described previously (Svistoonoff *et al.*, 2007; Ticconi *et al.*, 2009; Müller *et al.*, 2015). Seeds were surface sterilized with chlorine gas. The growth medium contained 5mM KNO_3_, 2.5mM KH_2_PO_4_, 2mM MgSO_4_, 2mM Ca(NO_3_)_2_, 50 µM Fe^3+^-EDTA, 70 µM H_3_BO_3_, 14 µM MnCl_2_, 0.5 µM CuSO_4_, 1 µM ZnSO_4_, 0.2 µM Na_2_MoO_4_, 10 µM CoCl_2_, and 5g l^–1^ of sucrose, pH 5.6 (Lincoln *et al.*, 1990). For the –Pi medium, KH_2_PO_4_ was omitted and the pH readjusted. The agar medium contained 0.75% (w/v) Phyto agar (Duchefa, Haarlem, The Netherlands), which was routinely purified as described elsewhere (Ward *et al.*, 2008).

### Hydroponic culture system

Liquefied agar medium (125 µl; 2.5mM Pi if not stated otherwise) was pipetted into each well of a 96-well PCR plate (Brand, Wertheim, Germany). After solidification, about 2mm of the bottom of each well was clipped off with sterilized scissors. One seed was placed with a toothpick on top of the agar of each well. Control wells (=blanks) received no seeds. The 96-well PCR plate was placed on top of a 96-deep-well block (Qiagen, Hilden, Germany) containing 1.7ml of liquid medium per well. The cut bottom of the 96-well PCR plate was immersed about 2–3mm into the medium. To complete the assembly, the insert of a 96-pipette tip (0.1–30 µl) box (VWR International, Darmstadt, Germany) was mounted on the PCR plate and deep-well block, and a sterile lid of a 96-well plate (Greiner Bio-One, Frickenhausen, Germany) was placed on top. All parts were fixed using leucopore tape (Duchefa). The blocks were incubated in the dark for 2 d at 4 °C for stratification and thereafter placed on a rotary shaker (80rpm) in a growth chamber at 22 °C under illumination for 16h daily (170 µmol s^–1^ m^–2^; Osram LumiluxDeLuxe Cool Daylight L58W/965, Osram, Augsburg, Germany). After 5 d, when the primary roots penetrated the agar plug, the PCR plate containing the seedlings was transferred to a new 96-deep-well block containing 1.7ml per well of fresh sucrose-free +Pi or –Pi medium so that each root was immersed into the liquid. After another 7 d of growth under the same conditions as for the first growing period, the hydroponic system was disassembled, and the medium was collected and stored at –80 °C. Roots were cut at the base of the 96-well PCR plate, straightened, and their length recorded with a ruler. Finally, root samples were placed into 2ml Eppendorf tubes, flash frozen in liquid nitrogen, and stored at –80°C until further processing.

### Non-targeted metabolite profiling of semi-polar compounds

The medium of eight wells was combined (about 13.5ml), spiked with 100 nmol 2,4-dichlorophenoxy acetic acid (Duchefa), and passed through Bond Elut C18 solid-phase extraction cartridges (500mg per 3ml; Agilent, Waldbronn, Germany) equilibrated with 1ml of methanol followed by 1ml of 1% (v/v) formic acid in water. The resin was washed with 1ml of water, and analytes were eluted with 1ml of 2% (v/v) formic acid in methanol. Eluates were evaporated under vacuum in a Savant SC210A SpeedVac Concentrator at 45 °C (ThermoFisher Scientific, Waltham, MA, USA) and the dry residues were taken up in 100 µl of 30% (v/v) of methanol. Non-targeted metabolite profiling was performed using an ultraperformance liquid chromatography (UPLC) electrospray ionization quadrupole-time-of-flight MS system (Waters, Eschborn, Germany; Bruker Daltonics, Bremen, Germany) as described previously (Strehmel *et al.*, 2014).

Each experiment consisted of at least three replicates per genotype and treatment, and three experiments were performed in a 10-month interval. Data evaluation and calculation were performed independently for each experiment. Within each experiment, replicates for each treatment and genotype were grouped, and data were processed using the R package XCMS (Smith *et al.*, 2006) as described previously (Strehmel *et al.*, 2014) with an intensity threshold of 1000 and a minfrac value of 1. The intensities of the resulting features (*m/z* retention time pairs) were log_10_ transformed and exported to MultiExperiment Viewer (Saeed *et al.*, 2003) for hierarchical cluster analysis (Pearson correlation, average linkage). Two-factor ANOVA (factor assignments: genotype and treatment; *P*<0.05) was applied before data clustering. For the creation of Venn diagrams, the following procedures were applied: (i) Student’s *t*-test (two-tailed, equal variance) was performed to estimate differences in signal intensities between +Pi and –Pi samples for each genotype, and features displaying a value of *P*>0.05 were omitted: (ii) the remaining features exhibiting a <1.5-fold difference in median intensities between +Pi and –Pi treatments were omitted; (iii) steps 1 and 2 were applied to the signal intensities of samples and controls, and all features with signal intensities that were <1.5-fold higher (*P*>0.05) in samples compared with the controls were discarded. After filtering, overlapping features (*m/z* values <0.01 deviation, retention time <5s deviation) across all three experiments were determined and selected for subsequent analysis. For high-resolution (HR)-MS/MS analysis, root exudates harvested from 96 wells (about 150ml) were processed as described above and reconstituted in a final volume of 100 µl. HR-MS/MS spectra were recorded using segmented UPLC-MS/MS runs (maximal two MS/MS spectra per segment) at a collision energy of 10eV according to Strehmel *et al.* (2014).

### Preparation of root extracts for targeted metabolite profiling

One root per replicate (1–5mg) was ground using a 5mm steel ball in a bead mill at 25s^–1^ for 50s. The resulting powder was extracted by vigorous shaking for 20min with 100 µl of 70% (v/v) methanol containing 2 nmol each of 4-methyl-umbelliferon and norvaline as well as 5 nmol each of [2,2,4,4-^2^H]citric acid, [2,3,3-^2^H)]malic acid, and [2,2,3,3-^2^H)]succinic acid as internal standards. After two centrifugations at 10 000*g* for 5min each, the resulting supernatant was stored at –80 °C until further processing.

### Targeted coumarin profiling

Root exudates harvested from single wells (about 1.7ml) were spiked with 50 pmol of 4-methyl-umbelliferon as an internal standard, and applied to a 96-well HR-X solid-phase extraction plate conditioned with 1ml of methanol followed by 1ml of water. The 96-well HR-X plate was prepared by dispensing 50mg of dry HR-X resin (Macherey-Nagel, Düren, Germany) into each well of a 96-well filter plate. In all steps, the liquid was passed through the resin by centrifugation at 250 *g* for 5min. After loading the samples, the resin was washed with 1ml of water, and coumarins were eluted with 1ml of methanol. After evaporation, the residue was resuspended in 20% (v/v) methanol and transferred to autosampler vials for LC-MS/MS analysis. Root extracts were transferred to autosampler vials without further processing.

LC-MS/MS analysis was performed using an Agilent 1290 LC system connected to an API 3200 triple quadrupole mass spectrometer by a TuboIon ion source (AB Sciex, Darmstadt, Germany). Coumarins were separated on a Nucleoshell C18 column (2.6 µm, 50×3mm; Macherey-Nagel) at 35 °C at a flow rate of 500 µl min^–1^ using 0.02% (v/v) acetic acid in water or acetonitrile as eluents A and B, respectively. After an initial hold at 5% B for 0.5min, the percentage of B was linearly increased to 45% within 4.5min, and thereafter to 95% B within 0.5min. After 1min at 95% B, the starting conditions were restored within 0.5min, and the column was allowed to re-equilibrate for 1.5min. The ion source was operated in the positive mode at a curtain gas pressure of 30 psi, an ion source voltage of 4500V, a temperature of 450 °C, and a sheath and desolvation gas pressure of 50 psi. Data were acquired in the scheduled multiple reaction monitoring mode (Q1 and Q3 set at unit resolution) with a window of 60s and a target scan time of 0.2s. Quantifier and qualifier transitions for each compound as well as compound-specific instrument parameters are shown in Supplementary Table S1 at *JXB* online.

Peak areas were calculated using the IntelliQuant algorithm of the Analyst 1.6.2 software (AB Sciex) and manually adjusted if necessary. All subsequent calculations were performed with Excel (Microsoft Office Professional Plus 2010), using the peak areas of 4-methyl-umbelliferon as the internal standard for quantification.

Compound-specific parameters were optimized by infusion of each coumarin standard (1 pmol µl^–1^ in 25%, v/v, acetonitrile) into the ion source via a syringe pump (Hamilton OEM Syringe Pump, 1ml syringe; Hamilton Bonaduz AG, Bonaduz, Switzerland) at a flow rate of 10 µl min^–1^. Ion source parameters were set to: curtain gas 10 psi, ion spray voltage: 4000V, ion source temperature: room temperature, nebulizing gas: 30 psi, and drying gas: 0 psi (=off).

### Amino acid analysis

Amino acids were analyzed as described by Ziegler and Abel (2014) using the root extract described above.

### Organic acid analysis

Ten microliters of root extract was evaporated to dryness, methoxylated with 20 µl of 20mg ml^–1^ of methoxyamine in pyridine for 1.5h at room temperature, and silylated for 30min at 37 °C with 35 µl of Silyl 991. Gas chromatography (GC)-MS/MS analysis was performed using an Agilent 7890 GC system equipped with an Agilent 7000B triple quadrupole mass spectrometer operated in the positive chemical ionization mode (reagent gas: methane, gas flow: 20%; ion source temperature: 230 °C). One microliter was injected [pulsed (25 psi) splitless injection, 220 °C] and separations were performed on an Rxi^®^-5ms column (15 m×0.25mm, 0.25 µm; Restek GmbH, Bad Homburg, Germany) using helium as a carrier gas (2.39ml min^–1^). The initial temperature of 70 °C was held for 1min, followed by increases at 9 °C min^–1^ to 150 °C and 30 °C min^–1^ to 300 °C. The final temperature of 300 °C was held for 5min. The transfer line was set to a temperature of 250 °C, and N_2_ and He were used as collision and quench gasses, respectively (1.5 and 2.25ml min^–1^). Data were acquired by multiple reaction monitoring using compound-specific parameters (see Supplementary Table S2 at *JXB* online) with Q1 and Q3 set to unit resolution. Peak areas were automatically integrated using the Agile algorithm of the MassHunter Quantitative Analysis software (version B.06.00) and manually adjusted if necessary. All subsequent calculations were performed with Excel, using the peak areas of the respective internal standards for quantification.

### Lignin staining

Roots were stained for 10min in 20% HCl containing an ethanolic solution of phloroglucinol (1% v/v) and observed under a Nikon SMZ1000 or Zeiss AxioImager microscope.

## Results

### Split-root hydroponic culture system

To monitor locally controlled root exudates in response to Pi limitation, a sterile hydroponic culture system was required that met the following criteria: it should (i) separate shoots and roots during hydroponic culture to eliminate shoot-derived metabolites; (ii) recapitulate reduction of primary root length as the most prominent phenotypic response to Pi deficiency; (iii) support labor-saving transfer of seedlings from Pi-replete to Pi-deplete liquid medium; and (iv) permit processing of many replicates per sample with minimal space requirements. We established a hydroponic culture system that met these requirements and allowed for the discrimination of local and systemic Pi starvation responses.

The 96-well hydroponic culture system is shown in Supplementary Fig. S1 at *JXB* online. The insert of the pipette tip box, placed on top of the preassembled PCR plate (filled with agar medium) and deep-well block (filled with liquid medium), is necessary to provide sufficient head space for shoot development underneath the cover lid. After 5 d of seed germination and seedling establishment in each well, the primary root penetrated the agar plug and elongated for a few millimeters into the liquid medium. At this stage, transfer of the roots to the medium of the designated composition was easily and quickly achieved by simply transferring the PCR plate to a new 96-deep-well block, which was not possible if plants were grown for more than 5 d. The 96-well design requires little space, and root exudates under different growth conditions can be collected because each root system develops in a separate compartment (deep well). Using this hydroponic culture system, the Arabidopsis wild-type (Col-0) and mutant (*pdr2* and *lpr1 lpr2*) lines grew and developed well with an average seed germination rate of 70–85%. Because of reduced germination rates in the absence of Pi or sucrose in the agar plugs, the agar medium contained Pi and sucrose. Sucrose was omitted from the second growth phase in the liquid medium, which facilitated extraction and chemical analysis of root exudates.

**Fig. 1.**
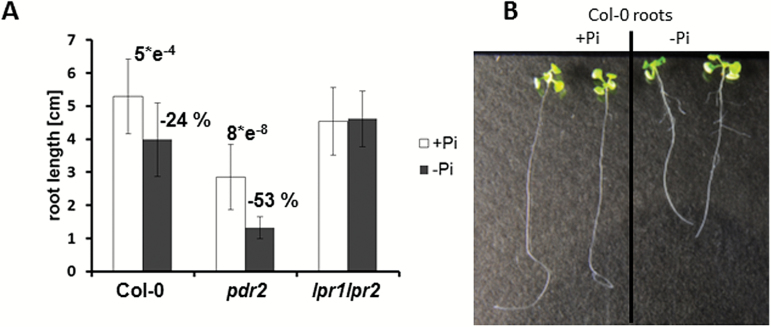
Root growth in the split-root hydroponic culture system. (A) Primary root length after seed germination and penetration of the +Pi (2.5mM) agar plug (5 d) and subsequent transfer for 7 d to +Pi (2.5mM) or –Pi (no Pi added) liquid medium. Error bars denote SD (*n*=16–20). The number above the bars for wild-type and *pdr2* roots indicate the *P* values of Student’s *t*-test (two-tailed, two sample equal variance) between +Pi and –Pi treatment. Data from one representative out of three independent experiments are shown. (B) Roots of wild-type plants after transfer to +Pi (left) and –Pi (right) liquid medium.

Although several mutants with defects in the induction of systemic Pi deficiency responses have been described (Yang and Finnegan, 2010; Chiou and Lin, 2011), the modular split design of the hydroponic culture system offers the advantage of studying local root responses to Pi deficiency independently of systemic Pi status. Whereas high Pi levels in the upper compartment (agar plug) were expected to suppress systemic Pi deficiency responses, Pi limitation in the lower compartment (liquid medium) likely elicited local responses. Indeed, inhibition of primary root growth, a typical local response to Pi limitation, was observed after seedling transfer to low-Pi liquid medium, despite the exposure of the upper root system to high-Pi agar (Fig. 1A). The primary root length of the wild-type and hypersensitive *pdr2* line was reduced by 24 and 53%, respectively, whereas the insensitive *lpr1 lpr2* double mutant was not affected. However, *pdr2* seedlings developed shorter roots under high-Pi conditions when compared with experiments performed on agar plates. Closer inspection of wild-type roots under Pi-replete conditions revealed long primary roots with some shorter lateral roots emerging at the base of the primary roots. Under Pi-deplete conditions, more and longer lateral roots were formed (Fig. 1B), which was consistent with root phenotypes observed on solid medium.

**Fig. 2.**
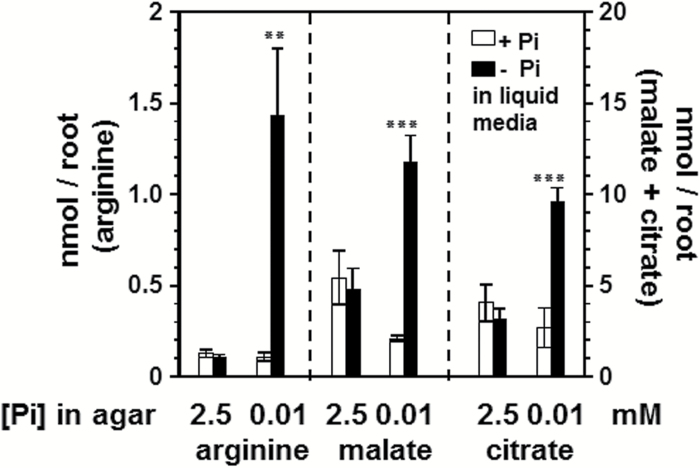
Arginine, malate, and citrate levels in roots of wild-type (Col-0) plants. Seeds were germinated and grown on agar plugs containing either 2.5mM or 0.01mM Pi on top of liquid medium containing 2.5mM Pi. After 5 d, the top PCR plate was transferred to a 96-deep-well plate containing +Pi (2.5mM Pi) or –Pi (no Pi added) liquid medium for another 7 d. Error bars denote SD (*n*=4). Data from one representative out of three independent experiments are shown. Significance analysis between + Pi and –Pi treatment was performed by Student’s *t*-test (two-tailed, equal variance): **P≤ 0.01, ***P≤0.001.

To confirm suppression of systemic responses, we analyzed the level of three primary metabolites known to accumulate in roots upon Pi deficiency in a PHR1-dependent fashion, i.e. arginine, malate, and citrate (Morcuende *et al.*, 2007; Pant *et al.*, 2015). When the upper root section was exposed to 2.5mM Pi in the agar, we could not detect an increase in arginine, malate, or citrate in the lower part of the roots although they were exposed to Pi-deficient conditions (Fig. 2). On the other hand, when the agar plugs were supplied with only 10 µM Pi, a strong increase of these metabolites in roots was observed. This indicated that the systemic Pi deficiency response was suppressed when high Pi was applied to the upper part of the roots, whereas the local response was still detectable.

### Non-targeted profiling of apolar metabolites

**Fig. 3.**
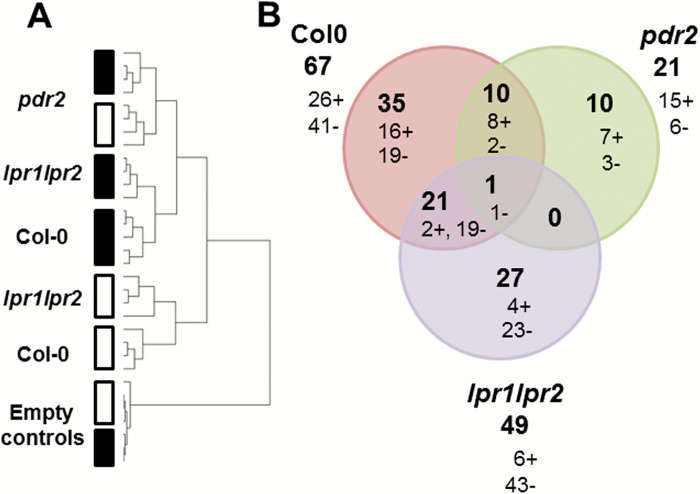
(A) Sample tree of hierarchical cluster analysis (Pearson correlation, average linkage) after two-factor ANOVA (factor assignments: genotype and treatment; *P*<0.05) for the non-targeted metabolite profiling datasets from experiment 3. White and black boxes denote +Pi (2.5mM) and –Pi (no Pi added) treatments, respectively. (B) Venn diagram showing the total number of differential features in root exudates for each genotype after +Pi (2.5mM) and –Pi (no Pi added) treatment and the number of overlapping features, as well as the number of genotype-specific features. The + and – signs after each number refer to higher intensities under +Pi and –Pi conditions, respectively. The numbers represent the overlaps between all three independent experiments. (This figure is available in color at *JXB* online.)

Initial LC-MS analyses showed that pooling of exudates from eight plants (medium of eight deep-wells) into a single sample was sufficient to detect about 4000 features (*m*/*z* retention time pairs). We performed three independent experiments, each consisting of at least three replicates (medium from eight pooled plants each) per genotype and treatment. Datasets that did not pass quality control after LC-MS runs were omitted (retention time shift >5s and intensity deviation >30% of the internal standard, 2,4-dichlorophenoxy acetic acid). The total number of datasets for each genotype and treatment in each experiment are listed in Supplementary Table S3 at *JXB* online. After data processing (see Material and methods), we obtained a total of 3200–9500 features per experiment, which comprise 2300–6800 features and 890–2700 features for the positive and negative ionization modes, respectively (Supplementary Data S1 and S2 at *JXB* online). Hierarchical cluster analysis of the data showed that the genotypes and treatments were clearly distinguishable based on their metabolite profiles (Fig. 3A). The distinction between +Pi and –Pi treatments predominated the distinction between wild-type and *lpr1 lpr2* genotypes, while the root exudate metabolite profiles of *lpr1 lpr2* and wild-type plants grown under –Pi were more similar to *pdr2* plants. Compared with wild-type and *lpr1 lpr2*, the effect of Pi deficiency on root exudates of *pdr2* was less pronounced.

To identify genotype-specific compounds that differentially accumulate in root exudates upon Pi deficiency, we determined the number of features exhibiting different abundances in root exudates after +Pi or –Pi exposure. As shown in Fig. 3B, when compared with the +Pi treatment, we detected 67 differential features (>1.5-fold difference, *P*<0.05) for wild-type roots exposed to Pi limitation, of which 26 and 41 features were of lower and higher abundance, respectively.

For *lpr1 lpr2* root exudates, 43 of the 49 differential features were of higher abundance after –Pi treatment. Exudates of *pdr2* roots revealed the least number of differential features (21), most of which (15) displayed decreased intensity after Pi deficiency. The wild type shared 22 and 11 features with *lpr1 lpr2* and *pdr2*, respectively. Only one feature was shared between all three genotypes, which showed higher abundance under Pi-deplete conditions (Fig. 3B). Conversely, several genotype-specific features were observed for the wild-type (35), *lpr1 lpr2* (27), and *pdr2* (10) lines.

### Identification of features with decreased intensity after Pi deprivation

We further analyzed all 104 detected differential features by high-resolution collision-induced dissociation mass spectrometry. Of the 104 differential features, about one-third (37) displayed lower abundance in Pi-limiting conditions. Among these, eight features were shared between wild-type and *pdr2*, and two features were common between wild type and *lpr1 lpr2*, whereas 16, seven, and four features were unique to wild-type, *pdr2*, and *lpr1 lpr2* root exudates, respectively (Fig. 3B). We obtained mass spectra for 14 features.

Interestingly, 11 features (including one isotope) showed mass spectra consistent with the fragmentation patterns of coumarins (Table 1, Supplementary Data S3 and Supplementary Fig. S2 at *JXB* online). Based on an authenticated standard, two features (*m*/*z* 377.08; *m*/*z* 193.05, both at 197s) were identified as the sodium adduct and the in-source aglycone fragment of scopolin, respectively. Seven features with retention times at 203s and 204s likely represented one compound. In the negative ionization mode, we detected an [M-H]^–^ ion of *m*/*z* 223.02 together with a sodium formate and potassium formate adduct (*m*/*z* 374.96), as well as a fragment after loss of a ^•^CH_3_ radical (*m*/*z* 208). Under positive ionization, an [M+H]^+^ ion at *m*/*z* 225.04 together with a sodium adduct (*m*/*z* 247.01), and a fragment after loss of a ^•^CH_3_ radical (*m*/*z* 210.02) were detected. Collision-induced dissociation in negative and positive ionization mode yielded neutral losses of CO, CH_3_OH, CO_2_, H_2_O, and H_2_CO, as well as ^•^CH_3_ radical elimination. The fragments exhibited a mass increase by *m*/*z*=16 and *m*/*z*=32 compared with mass spectra of authentic fraxetin (hydroxyscopoletin) and scopoletin standards, respectively. Therefore, we tentatively annotated this compound as dihydroxyscopoletin. We also detected two signals showing two mass units less compared with the [M-H]^–^ and the [M-H-CH_3_]^•–^ ions of the tentative dihydroxyscopoletin (*m*/*z* 221.01 and *m*/*z* 205.98). The collision-induced dissociation mass spectrum yielded several fragments with *m*/*z* of two mass units less than those obtained for the tentative dihydroxyscopoletin, suggesting a coumarin-type compound.

**Table 1.** Features of decreased abundance in root exudates of wild type (Col-0) after Pi starvation Only features that presumably represent coumarins are shown.

*m*/*z*	t_r_ (s)	Ion	Elemental composition	Tentative annotation/ characterization^*a*^	Ratio +Pi:–Pi	SD (*n*=3)	*t*-test (+Pi vs –Pi)
193.05	197	[M+H]^+^	C_10_H_9_O_4_^+^	In-source aglycone fragment of scopolin	1.78	0.13	0.018
377.08	197	[M+H+Na]^+^	C_16_H_19_O_9_Na^+^	Na adduct of scopolin	1.73	0.06	0.015
205.98	203	[M-H]^–^	C_9_H_2_O_6_^–^	Dihydroxyscopoletin-H_2_-CH_3_	12.28	9.19	0.008
208.00	203	[M-H]^–^	C_9_H_4_O_6_^–^	Dihydroxyscopoletin-CH_3_	6.30	0.07	0.009
210.02	204	[M+H]^+^	C_9_H_6_O_6_^+^	Dihydroxyscopoletin-CH_3_	8.80	4.14	0.005
221.01	203	[M-H]^–^	C_10_H_5_O_6_^–^	Dihydroxyscopoletin-H_2_	5.63	1.99	0.012
223.02	203	[M-H]^–^	C_10_H_7_O_6_^–^	Dihydroxyscopoletin	11.06	3.66	0.008
225.04	204	[M+H]^+^	C_10_H_9_O_6_^+^	Dihydroxyscopoletin	8.08	1.16	0.007
226.04	204			Dihydroxyscopoletin isotope	12.54	4.50	0.011
247.01	204	[M+H+Na]^+^	C_10_H_9_O_6_Na^+^	Na adduct of dihydroxyscopoletin	9.27	5.13	0.019
374.96	203	[M-H+NaFA+KFA]^–^	C_12_H_11_O_10_NaK^–^	NaFA and KFA adduct of dihydroxyscopoletin	5.17	2.94	0.021

^*a*^ For comprehensive datasets and detailed descriptions of feature annotation and characterization, see Supplementary Data S3. For structures see Supplementary Fig. S2.

**Fig. 4.**
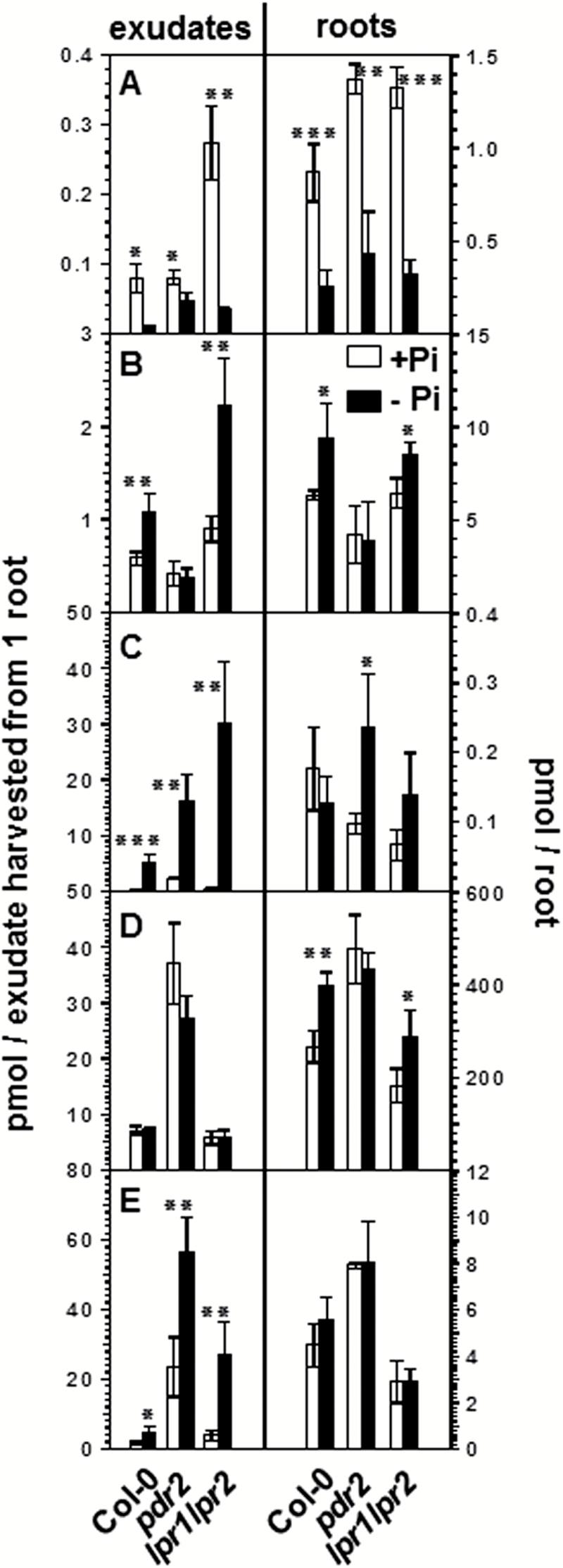
Coumarin content in exudates (left panels) and the corresponding roots (right panels). (A) Dihydroxyscopoletin (only exudates) and dihydroxyscopoletin glucoside (only roots). (B) Esculin. (C) Esculetin. (D) Scopolin. (E) Scopoletin. Error bars denote SD (*n*=4) from one representative out of three independent experiments. Significance analysis between + Pi and –Pi treatment was performed by Student’s *t*-test (two-tailed, equal variance): *P≤0.05, **P≤ 0.01, ***P≤0.001. For structures see Supplementary Fig. S2.

Coumarin exudation plays an important role in iron acquisition (Fourcroy *et al.*, 2013; Schmid *et al.*, 2014; Schmidt *et al.*, 2014), and Pi deficiency-induced changes in root morphology are dependent on iron availability (Ward *et al.*, 2008; Müller *et al.*, 2015). Therefore, we inspected the raw data for the signal intensities of other coumarins. Here, we noticed that several coumarins (such as esculin, esculetin, and scopoletin) just failed to comply with the stringent parameters of either >1.5 fold difference or *P*<0.05 between +Pi and –Pi treatments in one out of the three experiments. This prompted us to analyze coumarin levels in the hydroponic culture system using a targeted metabolite profiling approach. We quantified the content of scopoletin, esculetin, fraxetin, and of the tentative dihydroxyscopoletin together with the corresponding glucosides, i.e. scopolin, esculin, fraxin, and dihydroxyscopoletin glucoside, in root exudates and roots by LC-MS/MS using multiple reaction monitoring. For all genotypes, the content of both dihydroxyscopoletin and its glucoside decreased upon Pi deficiency in root exudates and roots (Fig. 4). Compared with *pdr2*, the decrease in the aglycone was more pronounced in wild-type and *lpr1 lpr2* exudates. However, the absolute amount of dihydroxyscopoletin in *lpr1 lpr2* root exudates was about 3-fold higher in Pi-replete conditions. In contrast to dihydroxyscopoletin and its glucoside, the content of other coumarins did not considerably change or rather increased upon Pi deficiency. Esculin as well as scopolin were predominantly present in roots, where their amounts increased by 30–50% upon Pi depletion in wild type and *lpr1 lpr2*, with no change in *pdr2*. In exudates, esculin content increased by factors of 1.8 and 2.4 in wild type and *lpr1 lpr2*, respectively, but scopolin content remained unchanged. Exudates of *pdr2* roots showed remarkably high amounts of scopolin, irrespective of the condition. No change in scopoletin content was observed in roots; however, it was strongly increased (2.5- to 7-fold) in exudates of all genotypes. Similar to scopolin, the amount of scopoletin was higher for *pdr2* root exudates. In roots, esculetin only accumulated significantly (*P*<0.05) in *pdr2* plants, whereas strongly increased esculetin levels were observed in root exudates of all genotypes. Exudates of *pdr2* exhibited the lowest increase due to high-level exudation of esculetin under Pi-sufficient conditions (Fig. 4).

### Identification of features with increased intensity after Pi deprivation

We detected 67 features that were increased in Pi-deplete conditions across all genotypes (Fig. 3B). Wild-type and *lpr1 lpr2* root exudates showed a similar number of features (41 and 43, respectively) of which 20 were shared, whereas only six features were observed for *pdr2*. The overlap of both genotypes with *pdr2* was low: only three features were shared with wild type and one was in common with *lpr1 lpr2*. The numbers of genotype-specific features were 19, three, and 23 for wild type, *pdr2*, and *lpr1 lpr2*, respectively.

Most interpretable fragmentation patterns resembled those of oligolignols, which are composed of coniferyl and sinapyl alcohol units after radical–radical coupling at different positions affording 8-*O*-4, 8-5, and 8-8 oligomers (Table 2, Supplementary Fig. S2 and Supplementary Data S4 at *JXB* online). Their MS/MS spectra exhibit coupling-type and subunit-specific fragments as well as consecutive losses of H_2_O and CH_2_O (Morreel *et al.*, 2010). Based on the spectra, five features could be assigned to three dilignols. One dimer did not solely consist of coniferyl alcohol units but contained ferulic acid as a monomer (*m*/*z* 353.10 at 362s). The only feature (*m*/*z* 327.12 at 276s) that was detected upon Pi deficiency in all genotypes could be characterized as a fragment of G(8-*O*-4)G after loss of H_2_O and CH_2_O, and also belonged to this group of dilignols. Eight features could be assigned to six trilignols, two of which were composed entirely of coniferyl subunits with different linkage types (*m*/*z* 571.22 at 288s [G(8-*O*-4)G(8-*O*-4)G] and *m*/*z* 553.21 at 341s [G(8-*O*-4)G(8-5)G]). The remaining trilignols consisted of coniferyl and sinapyl alcohol subunits of different composition and linkage types. On average, the oligolignols accumulated between 3-fold (*m*/*z* 553.21 at 376s) and 30-fold (*m*/*z* 583.22 at 367s) in roots exudates of Col-0 upon Pi deficiency. The features with *m*/*z* 327.12 at 344s and *m*/*z* 583.22 at 367s were also among the most intense signals detected in all experiments and samples under negative ionization conditions.

**Table 2.** Features of increased abundance in root exudates of wild type (Col-0) after Pi starvation Only features that presumably represent oligolignols are shown.

*m*/*z*	*t*_r_ (s)	Ion	Elemental composition	Tentative annotation/ characterization^*a*^	Ratio +Pi:–Pi	SD (*n*=3)	*t*-test (+Pi vs –Pi)
**327.12**	**276**	**[M-H]** ^**–**^	**C**_**19**_ **H**_**19**_ **O**_**5**_^**–**^	**G(8-*O*-4)G-H**_**2**_ **O-CH**_**2**_ **O**	**0.090**	**0.060**	**0.016**
195.06	281	[M-H]^–^	C_10_H_11_O_4_^–^	A^–^ fragment of β-arylether	0.200	0.080	0.005
571.22	288	[M-H]^–^	C_30_H_35_O_11_^–^	G(8-*O*-4)G(8-*O*-4)G	0.230	0.090	0.016
553.21	341	[M-H]^–^	C_30_H_33_O_10_-	G(8-*O*-4)G(8-5)G	0.070	0.040	0.001
327.12	344	[M-H]^–^	C_19_H_19_O_5_^–^	G(8-5)G-CH_2_O	0.110	0.030	0.001
328.13	344			G(8-*O*-4)G-H_2_O-CH_2_O isotope	0.100	0.070	0.001
339.12	344	[M-H]^–^	C_20_H_19_O_5_^–^	G(8-5)G-H_2_O	0.110	0.010	0.001
353.10	362	[M-H]^–^	C_20_H_17_O_6_^–^	G(8-5)-FA-H_2_O	0.280	0.170	0.030
583.22	367	[M-H]^–^	C_31_H_35_O_11_^–^	G(8-*O*-4)S(8-5)G	0.030	0.010	0.015
584.22	367			G(8-*O*-4)S(8-5)G isotope	0.040	0.010	0.007
585.23	366			G(8-*O*-4)S(8-5)G isotope	0.100	0.020	0.001
553.21	376	[M-H]^–^	C_30_H_33_O_10_^–^	G(8-*O*-4)G(8-8)S or G(8-*O*-4) S(8-8-G)-CH_2_O	0.350	0.090	0.009
613.23	402	[M-H]^–^	C_32_H_37_O_12_^–^	G(8-*O*-4)S(8-8)S	0.340	0.260	0.029
583.22	407	[M-H]^–^	C_31_H_35_O_11_^–^	G(8-*O*-4)G(8-8)S or G(8-*O*-4) S(8-8)G	0.100	0.030	0.001

^*a*^ For comprehensive dataset and detailed description of feature annotation and characterization, see Supplementary Data S4. The feature present in all genotypes is highlighted in bold. For structures see Supplementary Fig. S2. G, guaiacyl unit; S, syringyl unit; FA, ferulic acid

**Fig. 5.**
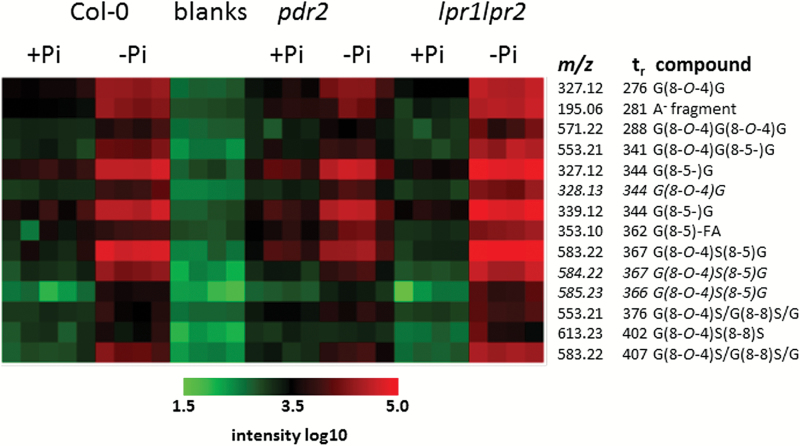
Heat map showing log_10_-transformed intensities for features tentatively assigned as oligolignols (Table 2). Data from the non-targeted metabolite dataset from experiment 3 are shown. For structures, see Fig. S2. G, guaiacyl unit; S, syringyl unit; FA, ferulic acid. “Blanks” refers to medium harvested from wells devoid of plants.

The heat map revealed increasing signal intensities in the order *pdr2*<wild type<*lpr1 lpr2* for most tentative oligolignols in Pi-limiting conditions (Fig. 5). The extent of accumulation after –Pi treatment was more pronounced for *lpr1 lpr2* when compared with wild-type and *pdr2* root exudates. Indeed, the abundance of oligolignols under Pi-sufficient conditions was 2-fold and 3-fold higher in *pdr2* exudates compared with wild type and *lpr1 lpr2*, respectively. However, under Pi-deficient conditions, *lpr1 lpr2* exudates contained 2.5-fold and 3.5-fold higher levels relative to wild type and *pdr2*, respectively. Consequently, the average increase in oligolignol abundance in exudates after transfer to Pi-deficient medium was 22-fold for *lpr1 lpr2*, 6-fold for wild type, and 3-fold for *pdr2* seedlings. With the exception of the signal with *m*/*z* 613.23 at 402s [possibly G(8-*O*-4)S(8-8)S], which did not change in *pdr2* exudates, all other lignin oligomers accumulated upon Pi deficiency in all genotypes, although to different extents. Of all the putative lignin oligomers, the feature *m*/*z* 583.22 at 367s [presumably G(8-*O*-4)S(8-5)G] showed the highest accumulation in *lpr1 lpr2* (68-fold) and wild type (30-fold), whereas for *pdr2* the signals with *m*/*z* 339.12 and *m*/*z* 327.12 at 344s [G(8-5)G] displayed the strongest increases at 5.5-fold and 4.9-fold, respectively.

### Lignification of root meristems in response to Pi limitation

**Fig. 6.**
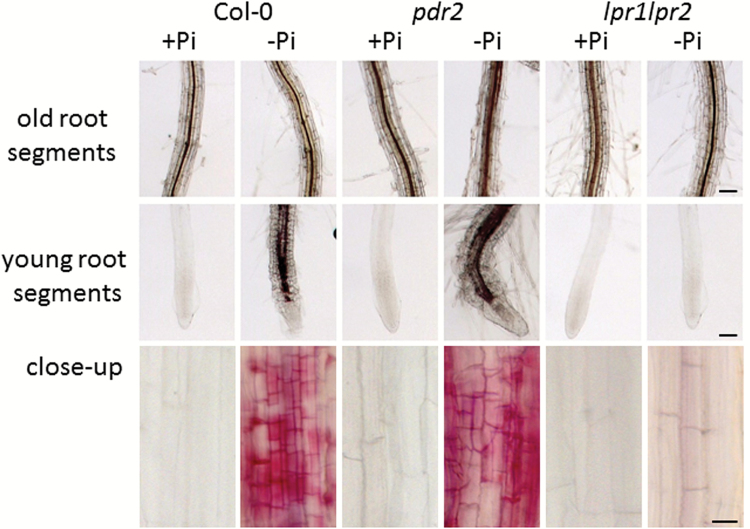
Phloroglucinol staining of roots after transfer to +Pi (2.5mM) or –Pi (no Pi added) medium for 7 d. Older parts of the roots (upper row), younger parts (middle row), and close-up view of the differentiation zone close to the root tips (lower row) are shown. Exudates were harvested from roots grown in +Pi (2.5mM) or –Pi (no Pi added) medium, respectively. Bars, 100 µm (upper/middle row); 20 µm (lower row).

Oligolignols are components of lignin, lignans, and neolignans. Lignin consists of numerous guaiacyl and syringyl units, whereas lignans and neolignans are primarily composed of two guaiacyl units (Morreel *et al.*, 2010; Vanholme *et al.*, 2010; Barros *et al.*, 2015). The detection of mainly trimers consisting of guaiacyl and syringyl units in root exudates prompted us to monitor lignification in primary roots (Fig. 6). Phloroglucinol staining of the vascular tissue in the upper region of the main root, close to the hypocotyl, was similar and consistent among all genotypes and treatments. In younger root segments, lignin staining was not observed for plants grown in Pi-sufficient medium. However, after transfer to Pi-deficient medium for 1 week, a patchy phloroglucinol staining was observed along the roots for the sensitive wild-type and *pdr2* lines. However, this staining pattern could not be observed in the insensitive *lpr1 lpr2* line.

## Discussion

Previous investigations have focused on root exudate metabolite profiling of polar compounds that are involved in Pi recycling and Pi acquisition strategies during the systemic response. In this study, we aimed to specifically analyze root exudation during the local response independently of the systemic response. We therefore developed a hydroponic system that separates both responses to limiting Pi. Root levels of arginine, malate, and citrate only increased if the entire root system was exposed to low Pi. However, if only the lower part of the root was exposed to liquid low-Pi medium, whereas the upper part grew in high-Pi agar, no accumulation of acids was detectable. Arginine, malate, and citrate belong to a group of several primary metabolites that accumulate upon Pi deficiency in a PHR1-dependent fashion, which is the main regulator of systemic Pi-starvation responses (Pant *et al.*, 2015). Therefore, we reasoned that the systemic response was suppressed by the high Pi concentration in the agar plugs, although it is difficult to draw a general conclusion based on the limited number of responses. However, we also did not observe purple pigmentation of shoots, which is indicative of anthocyanin accumulation and another indicator of a systemic Pi-deficiency response.

The genotypes investigated in our study are known to exhibit different local Pi starvation responses (Ticconi *et al.*, 2004; Svistoonoff *et al.*, 2007; Müller *et al.*, 2015). Whereas *pdr2* root growth displays hypersensitivity in low Pi, the *lpr1 lpr2* line is unaffected. The respective responses were also detectable under our hydroponic conditions, although the decrease in primary root length for wild type was less pronounced compared with experiments performed on agar plates. One reason might be diffusion of Pi from the +Pi containing agar plugs into the –Pi medium. Since the extent of the local response depends on the Pi concentration, diffusion of Pi into the low-Pi medium might increase the Pi content at the root tip, and thereby attenuate the response. Indeed, the Pi concentration in the Pi-deplete medium was approximately 50 µM at the time of harvest. Interestingly, *pdr2* seedlings already showed impaired primary root growth under Pi-sufficient conditions. The reason is unclear, but we observed that *pdr2* roots required more time to penetrate the agar plugs compared with wild-type or *lpr1 lpr2* plants. Despite this phenomenon, the decrease in primary root lengths after transfer to low-Pi conditions was stronger for *pdr2* compared with wild type or *lpr1 lpr2*. Although root growth behaved slightly different when compared with experiments on agar plates, we detected the local response at the lower part of the roots in our hydroponic system, while the systemic response was suppressed. For young seedlings, this is the only hydroponic system that has been developed so far allowing root exudate analysis upon Pi starvation without interference of systemic responses.

Root exudate profiles of plants exposed to Pi-sufficient and Pi-deficient media were clearly distinguishable for all genotypes. It is possible that the recorded profiles were caused by the release of endogenous compounds of cells that had been sloughed off the root rather than due to exudation. This effect would be more prominent for roots exposed to Pi-deplete medium, which undergo profound changes in root meristem morphology (Müller *et al.*, 2015). However, the exudate profiles of Pi-sufficient and Pi-deficient insensitive *lpr1 lpr2* plants were almost equally dissimilar as the exudate profiles of sensitive wild-type plants. Therefore, it is unlikely that the components contributing to the detected metabolite profiles were derived solely from endogenous compounds. This scenario might be different for *pdr2* plants. Irrespective of the treatment, the exudate profiles of *pdr2* plants were significantly different from wild type and *lpr1 lpr2*. Since *pdr2* roots are much shorter under Pi-deplete and Pi-replete conditions, which in case of Pi starvation is caused by profound changes in root meristem activity, the metabolite profiles might well be derived from endogenous compounds. However, despite the extremely shortened primary root, the decreased number of signals in root exudates of Pi-starved *pdr2* plants points to a disturbed exudation in this genotype. Impairment of exudation for *pdr2* was also evident considering the response to Pi deprivation. In contrast to wild type and *lpr1 lpr2*, the number of differential features upon Pi deficiency in *pdr2* root exudates was comparably low. Since we normalized the signal intensities to the number of plants rather than to fresh weight, this might be due to the decreased capacity of short roots to exude metabolites. However, we assume this not to be the case since (i) shortened primary roots in the wild type showed increased exudation and (ii) shortened primary root length was accompanied by increased lateral root length and number, thereby increasing the root surface.

Thus, *pdr2* plants are not only hypersensitive to low-Pi conditions with respect to root system architecture but are also impaired in Pi deficiency-induced root exudation. The *PDR2* gene encodes the single P5A-type ATPase, AtP5A/MIA (Jacobsen *et al.*, 2005; Ticconi *et al.*, 2009) and belongs to the large class of P-type ATPases that are mainly involved in ion, heavy metal, and possibly lipid transport (Baxter *et al.*, 2003). The precise biochemical function of the P5A-type ATPase is unknown, but several studies suggest its importance in the secretory pathway as well as in protein folding, processing, and targeting (Jakobsen *et al.*, 2005; Sørensen *et al.*, 2015). Also, the expression of several ABC-type and MATE transporters was strongly repressed in anthers of *male gametogenesis impaired anthers* (*mia*) plants harboring T-DNA insertions in the *PDR2* gene (Jakobsen *et al.*, 2005). Although experimental proof is needed, our data on impaired root exudation of *pdr2* further strengthen previous observations suggesting a role of PDR2 in regulating secretory processes.

Several features exhibiting decreased intensities in root exudates of Pi-deplete plants could tentatively be assigned to dihydroxyscopoletin, a coumarin. Metabolite profiling of root exudates exhibited strong increases in the content of the coumarins scopoletin, esculetin, fraxetin, and dihydroxyscopoletin during Fe deficiency in order to facilitate Fe acquisition (Fourcroy *et al.*, 2013; Schmid *et al.*, 2014; Schmidt *et al.*, 2014). The decrease of dihydroxyscopoletin after Pi deficiency is consistent with the antagonistic regulation of many genes by either Pi or Fe deficiency (Zheng *et al.*, 2009; Li and Lan, 2015). Furthermore, Fe accumulation promotes Pi deficiency-induced changes in root system architecture (Müller *et al.*, 2015). Thus, during Pi deficiency, decreased secretion of dihydroxyscopoletin, which accumulates most strongly upon Fe deficiency (Schmid *et al.*, 2014), might be a strategy by the plant to decrease Fe uptake and thereby restore primary root growth. However, the role of coumarins in Pi deficiency-induced Fe accumulation seems to be more complex. First, coumarin patterns are similar between insensitive *lpr1 lpr2* plants (no Fe accumulation) and sensitive wild-type and *pdr2* plants (Fe accumulation). Secondly, other coumarins with Fe mobilizing capabilities such as scopoletin and esculetin exhibited increased levels in root exudates. However, the secretion of other well-known facilitators for Fe uptake, especially organic acids, may mask the contribution of coumarins for Fe accumulation in Pi deficiency. As such, a conclusion as to whether or not there is a contribution of coumarin exudation to Fe accumulation upon Pi limitation cannot be drawn based on current results. Nevertheless, the decrease and increase in the levels of individual coumarins suggests differential regulation of coumarin biosynthetic pathway genes during Pi and Fe deficiency, where all coumarins have been reported to accumulate. In order to explore the molecular basis for the differential accumulation of individual coumarins under Pi deficiency, as well as to further explore the different Fe mobilizing capabilities of distinct coumarins, biosynthetic genes downstream of feruloyl-CoA 6'-hydroxylase, the committed step in coumarin biosynthesis (Kai *et al.*, 2008), need to be characterized.

Most of the signals exhibiting increased intensity in root exudates during Pi deficiency could tentatively be assigned to oligolignols. In addition to dimeric, coniferyl alcohol-derived lignans and neolignans, which might play roles as antioxidants and in plant defense, we also found trimeric lignin oligomers consisting of coniferyl and sinapyl alcohol, which are known to accumulate during lignification. Consistently, the accumulation of lignin oligomers in root exudates coincided with increased lignification in Pi-starved roots. Recently, an inverse relationship between the extent of lignification and oligolignol accumulation was observed in lignifying flax tissue (Huis *et al.*, 2012). Here, we also observed such an inverse relationship for oligolignol accumulation in root exudates and lignin deposition in roots after Pi deprivation. Furthermore, this effect correlated with the genotype-specific sensitivity toward Pi deprivation with respect to primary root growth. Oligolignol levels in root exudates were lower in sensitive wild-type and *pdr2* lines compared with the insensitive *lpr1 lpr2* line, whereas the opposite was observed for lignin deposition. It has long been assumed that lignin biosynthesis proceeds by peroxidase- and/or laccase-mediated oxidation of monolignols, which are first generated intracellularly and then transported to the cell wall (Barros *et al.*, 2015). Based on the occurrence of glucosylated oligolignols in Arabidopsis leaf vacuoles and labelling experiments, it has recently been suggested that combinatorial radical coupling of monolignols also occurs intracellularly and that the resulting oligolignols are incorporated into lignin after transport to the apoplast (Dima *et al.*, 2015).

The inverse correlation between oligolignol accumulation in root exudates and the extent of lignin deposition in roots suggests that such a scenario could also apply for roots, especially under Pi starvation. The absence of lignin and the strong accumulation of oligolignols in root exudates of *lpr1 lpr2* plants suggest a role for these proteins in Pi starvation-induced root lignification. LPR proteins are members of the multicopper oxidase protein family, which also includes laccases, and likely function as apoplastic ferroxidases (Hoegger *et al.*, 2006; Svistoonoff *et al.*, 2007, Müller *et al.*, 2015). Although more experiments are needed, our data suggest that the ferroxidase activity of LPR proteins may contribute to lignin deposition in cell walls of Pi-starved roots by polymerization of secreted oligolignols and may thereby be involved in primary root growth inhibition. Whether this is achieved by direct oxidation of oligolinols or by creation of a more oxidative environment remains to be elucidated.

In conclusion, the non-targeted metabolite profiling data of root exudates from three genotypes with different sensitivities toward Pi deficiency open several novel directions for future experiments. Thus, it would be worthwhile analyzing in more detail the role of PDR2 on root exudation, as the negative impact of the *pdr2* mutant on a large number of exuded compounds suggests a major factor controlling root exudation. Furthermore, the overlapping and contrasting effects on secretion of individual coumarins might represent an appropriate tool to further elaborate common or distinct signaling elements during Pi and Fe deficiency. Lastly, it will be interesting to further elucidate the role of oligolignol synthesis and lignification during Pi starvation-induced root growth inhibition, especially considering the function of LPR ferroxidases.

## Supplementary data

Supplementary data are available at *JXB* online.

**Fig. S1.** Assembly of the split-root hydroponic system showing shoots and roots at harvest after 12 d of growth.

**Fig. S2.** Structures of compounds.

**Table S1.** MRM parameters for targeted coumarin analysis.

**Table S2.** MRM parameters for targeted organic acid analysis.

**Table S3.** Number of non-targeted metabolite profiling datasets for each treatment and genotype.

**Data S1.** Non-targeted metabolite profiling data for all three experiments; negative ionization.

**Data S2.** Non-targeted metabolite profiling data for all three experiments; positive ionization.

**Data S3.** Characterization of features exhibiting decreased abundance in wild type (Col-0) root exudates after Pi deficiency.

**Data S4.** Characterization of features exhibiting increased abundance in wild type (Col-0) root exudates after Pi deficiency.

Supplementary Data
