# Supplementary material for: Non-targeted profiling of semi-polar metabolites in Arabidopsis root exudates uncovers a role for coumarin secretion and lignification during the local response to phosphate limitation
Source: J Exp Bot. 2015 Dec 17;67(5):1421–32. doi: 10.1093/jxb/erv539 (PMC4762384; doi:10.1093/jxb/erv539)
Supplement: Supplementary Data [file supp_67_5_1421__index.html]

Non-targeted profiling of semi-polar metabolites in Arabidopsis root exudates uncovers a role for coumarin secretion and lignification during the local response to phosphate limitation — Non-targeted profiling of semi-polar metabolites in Arabidopsis root exudates uncovers a role for coumarin secretion and lignification during the local response to phosphate limitation — Supplementary Data 

# Non-targeted profiling of semi-polar metabolites in Arabidopsis root exudates uncovers a role for coumarin secretion and lignification during the local response to phosphate limitation

## Supplementary Data

Data files

- supplementary\_figures\_S1\_S2\_tables\_S1\_S3.pdf - Supplementary Data
- Supplement\_dataset\_1.xlsx - Supplementary Data
- Supplement\_dataset\_2.xlsx - Supplementary Data
- Supplement\_dataset\_3.xlsx - Supplementary Data
- Supplement\_dataset\_4.xlsx - Supplementary Data
