# Supplementary material for: Non-targeted profiling of semi-polar metabolites in Arabidopsis root exudates uncovers a role for coumarin secretion and lignification during the local response to phosphate limitation
Source: J Exp Bot. 2015 Dec 17;67(5):1421–32. doi: 10.1093/jxb/erv539 (PMC4762384; doi:10.1093/jxb/erv539)
Supplement: Supplementary Data [file supp_erv539_supplementary_figures_S1_S2_tables_S1_S3.pdf]

Non-targeted profiling of semi-polar metabolites in Arabidopsis root exudates uncovers a role for coumarin secretion and lignification during the local response to phosphate limitation

Joerg Ziegler, Stephan Schmidt, Ranju Chutia, Jens Müller, Christoph Böttcher, Nadine Strehmel, Dierk Scheel, and Steffen Abel

Supplemental File

Supplementary Figure S1. Assembly of the split-root hydroponic system showing shoots and roots at harvest after 12 days of growth.
Supplementary Figure S2. Structures of compounds.

Supplementary Table S1. MRM parameters for targeted coumarin analysis.
Supplementary Table S2. MRM parameters for targeted organic acid analysis.
Supplementary Table S3. Number of non-targeted metabolite profiling datasets for each treatment and genotype.

Supplementary Fig. S1: Assembly of the hydroponic culture system

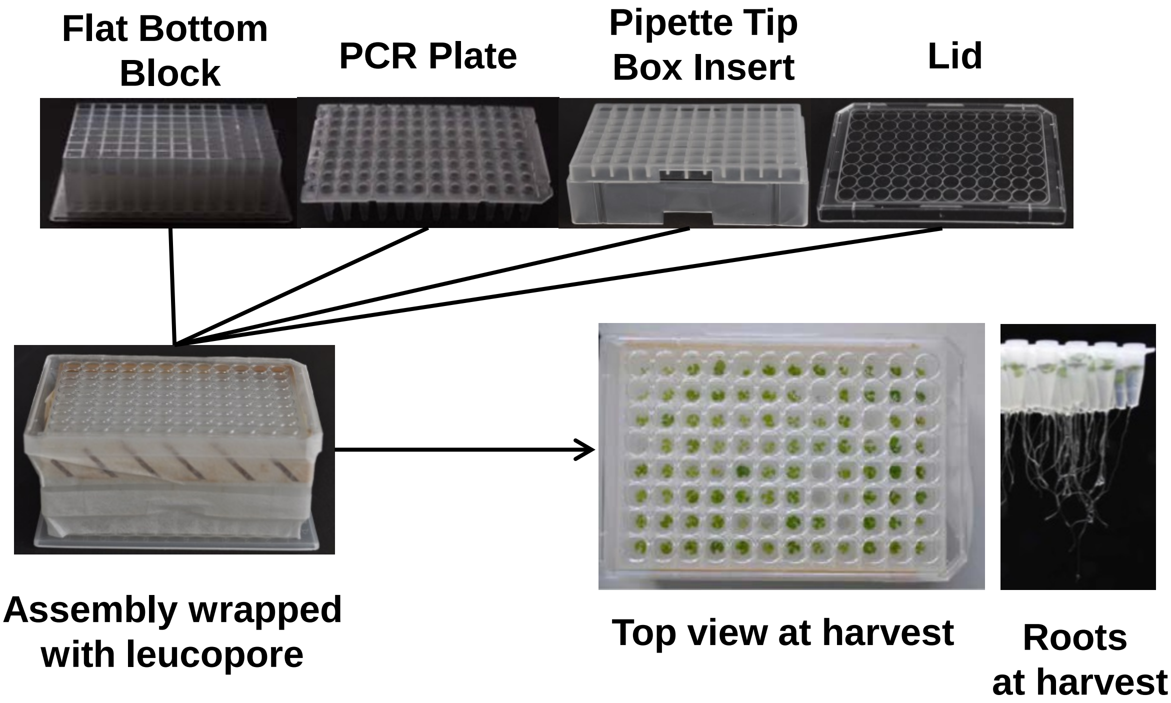

Supplementary Fig. S1: Assembly of the split-root hydroponic system showing shoots and roots at harvest after 12 days of growth.

Supplementary Fig. S2: Compound structures

A: Coumarins

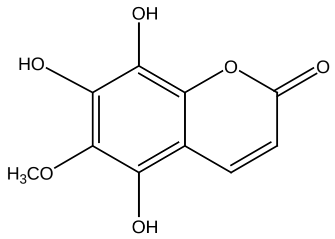

Dihydroxyscopoletin (proposal; position of hydroxy groups requires confirmation

m/z 374.96 at 203 s
m/z 208.00 at 203 s
m/z 223.02 at 203 s
m/z 247.01 at 204 s
m/z 210.02 at 204 s
m/z 225.04 at 204 s

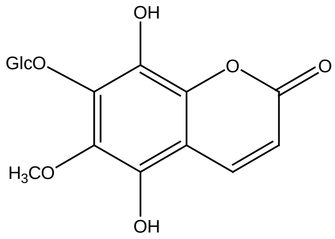

Dihydroxyscopoletin-Glucoside (proposal; position of hydroxy groups and glucose requires confirmation

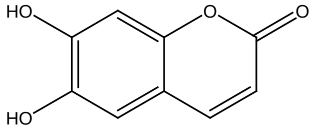

Esculetin

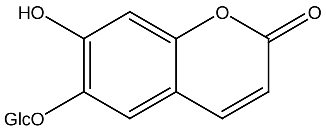

Esculin

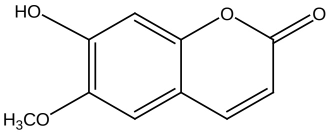

Scopoletin

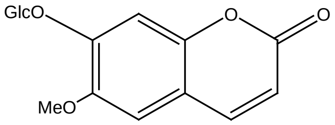

Scopolin

m/z 377.08 at 197 s
m/z 193.05 at 197 s

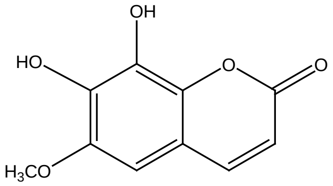

Fraxetin

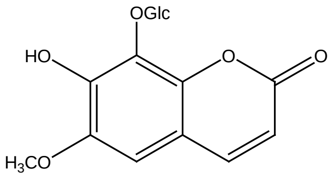

Fraxin

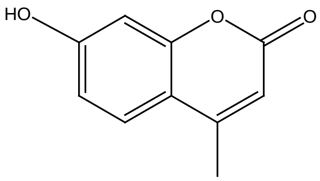

4-Methyl umbelliferon (internal standard)

B: Oligolignols

Dilignols

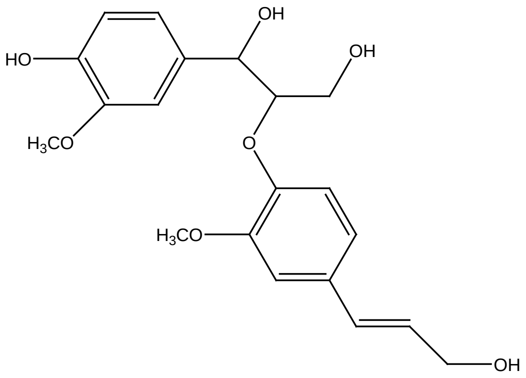

G(8-O-4)G

m/z 327.12 at 276 s

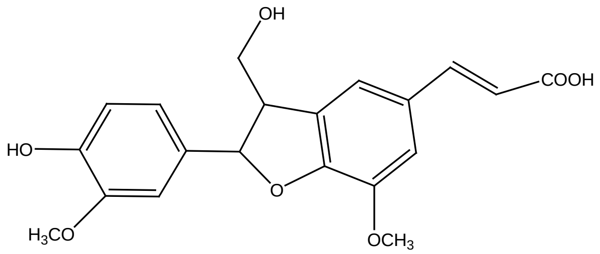

G(8-5)FA

m/z 353.10 at 362 s

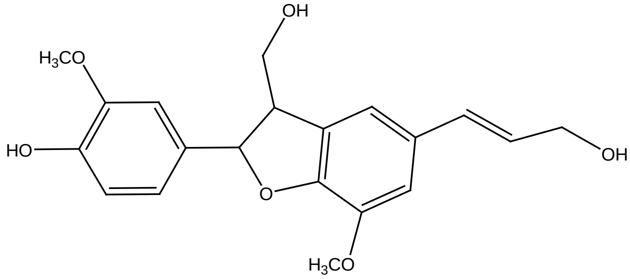

G(8-5)G

m/z 339.12 at 344 s

m/z 327.12 at 344 s

Trilignols

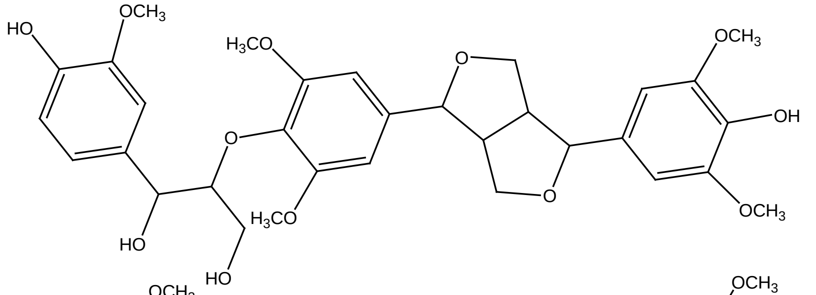

G(8-O-4)S(8-8)S

m/z 613.23 at 402 s

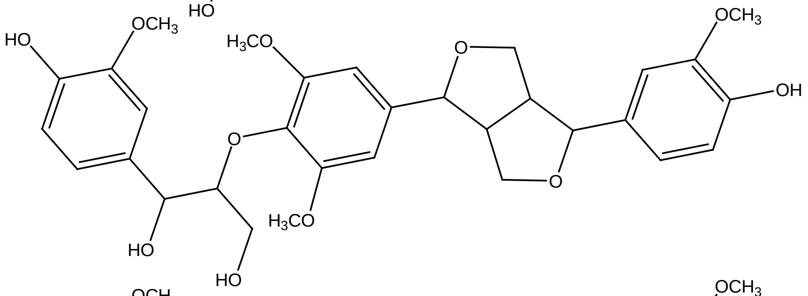

G(8-O-4)S(8-8)G

m/z 583.22 at 407 s or
m/z 553.21 at 376 s

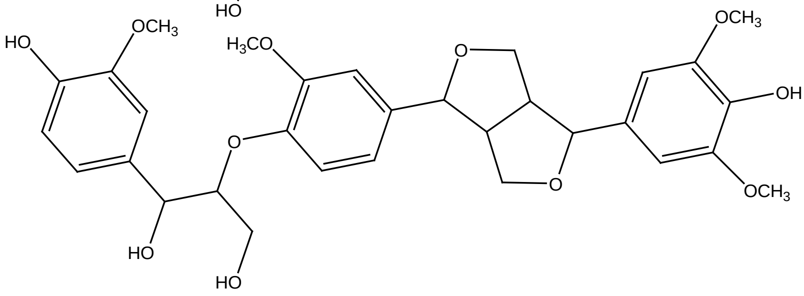

G(8-O-4)G(8-8)S

m/z 583.22 at 407 s or
m/z 553.21 at 376 s

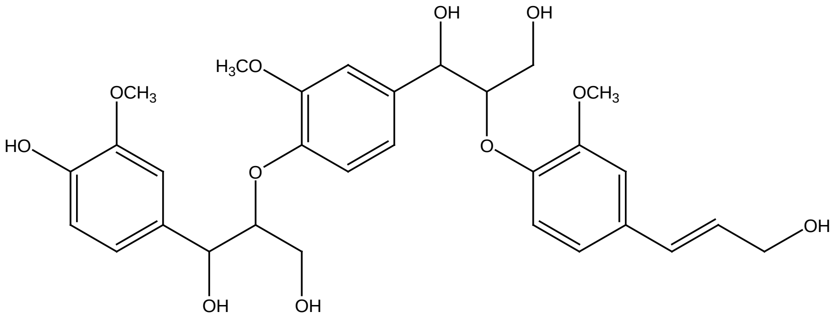

G(8-O-4)G(8-O-4)G

m/z 571.22 at 288

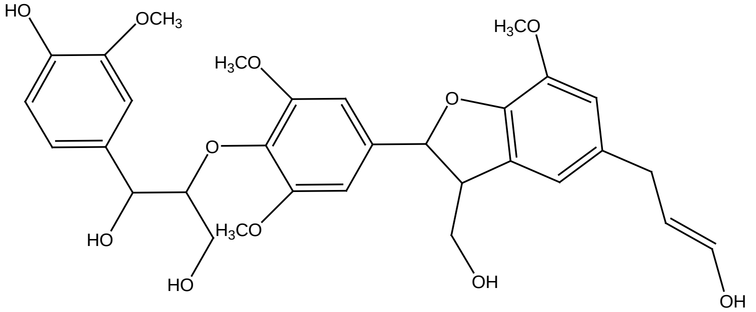

G(8-O-4)S(8-5)G

m/z 583.22 at 367

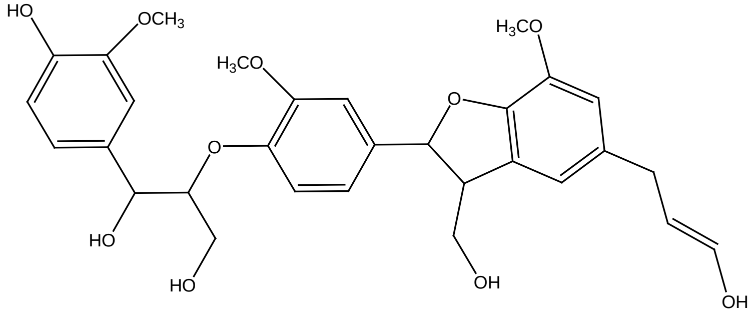

G(8-O-4)G(8-5)G

m/z 553.21 at 341

Supplementary Table S1 MS parameters for MRM-transitions

Coumarins	MRM transitions	Retention time, min	Declustering potential (DP), V	Entrance potential (EP), V	Cell entrance potential (CEP), V	Collision potential (CE), V	Cell exit potential (CEX), V
4-Methyl umbelliferon	**177→103**	3.8	51	3.5	10	33	4
	177→105		51	3.5	10	27	4
Esculetin	**179→123**	2.5	51	10.5	14	31	4
	179→133		51	10.5	14	27	4
Esculin	**341→179**	2.0	46	4.5	22	23	4
	341→123		46	4.5	22	59	4
Scopoletin	**193→133**	3.2	51	3.5	12	29	4
	193→178		51	3.5	12	29	4
Scopolin	**355→193**	2.4	26	4.5	24	19	4
	355→133		26	4.5	24	55	4
Dihydroxy scopoletin	**225→210**	2.5	25	9	15	30	4
	225→136		25	9	15	30	4
Dihydroxy scopoletin-glucoside	**387→225**	2.3	30	3	20	20	4
	387→210		30	3	20	45	4

Quantifier and qualifier transitions are indicated in bold and italics, respectively

SupplementaryTable S2 MS parameters for MRM-transitions

Organic acid	MRM transitions	Retention time, min	segment	ionization energy, eV	collision energy, eV
pyruvate	**190→100**	1.89	1	-135	5
	190→174				5
lactate	**235→191**	2.01			5
	235→219				5
oxalate	**235→163**	2.79			5
	235→219				5
succinate-D4	**267→177**	5.01	2	-135	5
	267→251				5
succinate	**263→173**	5.05			5
	263→247				5
fumarate	**261→245**	5.50			5
	261→171				10
malate	**351→233**	7.55	3	-135	5
	351→189				15
malate-D3	**354→236**	7.53			5
	351→192				15
α-ketoglutarate	**320→244**	8.65	4	-135	5
	320→230				10
aconitate	**391→211**	10.55			5
	391→301				15
citrate-D4	**485→276**	11.03	5	-135	5
	485→367				15
citrate	**481→273**	11.04			5
	481→363				15
gluconate	**629→539**	11.88			5
	629→369				15

Quantifier and qualifier transitions are indicated in bold and italics, respectively

Supplementary Table S3:

Number of datasets for each genotype and treatment

Non-targeted metabolite profiling of root exudates

experiment	date	ionization	Col0		*lpr1lpr2*		*pdr2*		control	
			+P	-P	+P	-P	+P	-P	+P	-P
1	2012-09-11	positive	2	3	3	3	3	3	3	3
		negative	3	3	3	3	3	3	3	3
2	2013-04-09	positive	5	5	5	5	5	5	4	5
		negative	5	5	3	5	5	5	5	5
3	2013-08-13	positive	5	4	4	4	4	4	3	5
		negative	5	4	4	4	4	4	3	5
	Σ	positive	12	12	12	12	12	12	10	13
		negative	13	12	10	12	12	12	11	13
